# Where are the cores in this thing? …and what are they computing? (with apologies to Larry Abbott)

**DOI:** 10.1007/s10827-021-00809-1

**Published:** 2022-02-04

**Authors:** Daniel Gardner

**Affiliations:** Departments of Physiology & Biophysics, Neurology, and Neuroscience, Weill Cornell Medical College, New York, NY 10065 USA

## Evolution conserves neural components and connectivity—does this extend to computations and circuitry?

**Fig. 1:**
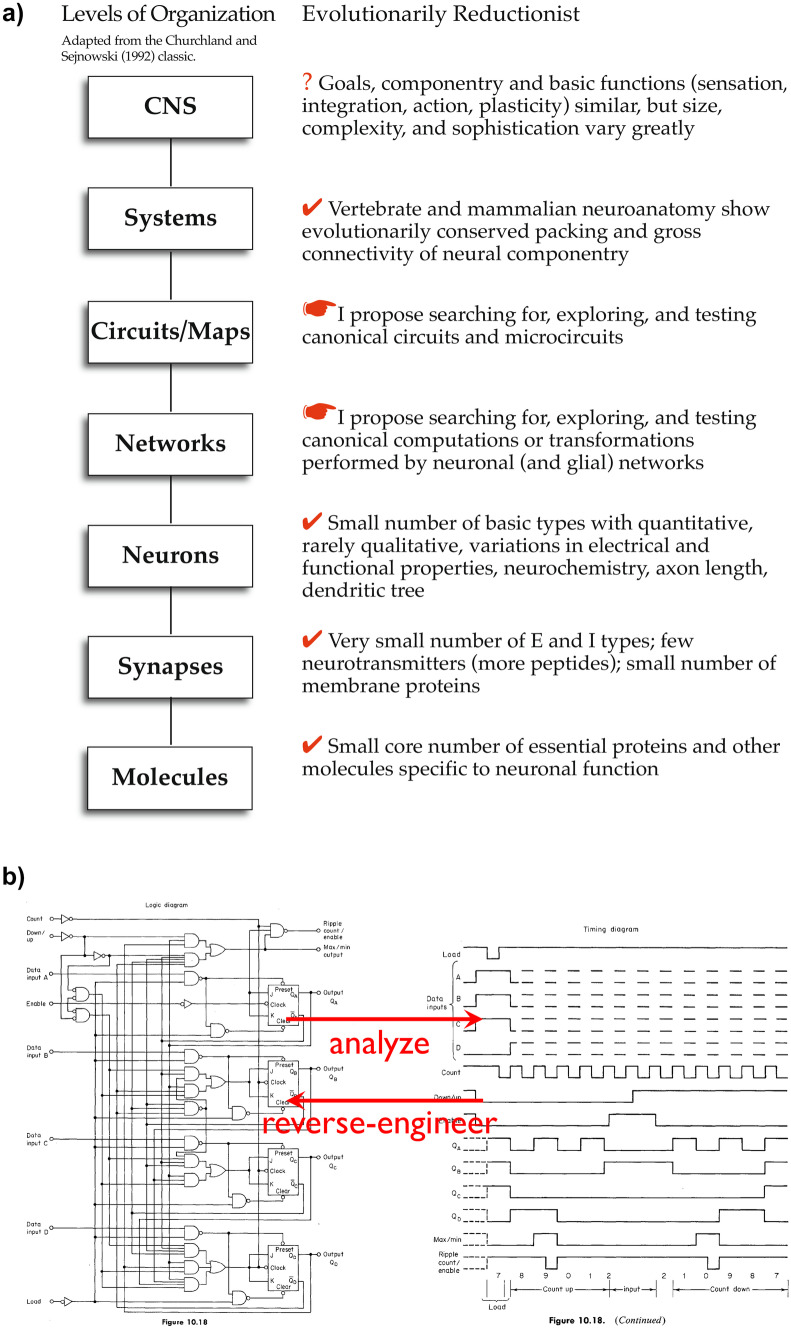
1a: Which of the components and functions in the classic levels defined by Churchland and Sejnowski (1992) are evolutionarily conserved? At lower levels, molecules, neurotransmitters, membrane proteins, synapse functions, and excitatory and inhibitory neurons are conserved, as are large-scale mammalian neuroanatomy at the top. But in between, this Perspective asks, are circuits, microcircuits and the computations and transforms they mediate similarly conserved? Is there a small number of canonical types? 1b: For electronic circuits, both digital as shown and analog, function can be predicted from component properties and circuit connectivity. Probes give data that permit reverse-engineering a circuit from recordings. This is not yet possible for BNNs. From Morris and Miller (1971)

In contrast to the broad diversity of the structure and function of life forms, nervous systems across species and even phyla are assembled from a remarkably similar set of components. Essentially all nervous systems use evolutionarily-conserved small molecules, neurotransmitters, proteins, excitatory and inhibitory neurons, and synapses. At higher levels, vertebrate and mammalian neuroanatomy conserves connectivity and functional role of nuclei, layers, and regions. At still higher levels, the goals and functions of all nervous systems are similar. Neural circuits have evolved to learn and to perform relevant pattern recognition, classify inputs, and make decisions. But at intermediate levels: are neural circuits and the computations they perform evolutionarily conserved? Is there a small number of canonical identifiable micro- and meso-circuit computations or algorithms that can be identified and verified (Fig. 1a)? We do not yet have the data to answer these questions. A provisionally useful assumption is that some computations—arithmetic, logical, transformative, and more—are performed by local circuits, and still others arise from the interconnections of such computationally-functional assemblies via both shorter and longer axons.

Evolutionary parallels motivate this Perspective, but there is another, even more fundamental, unknown. In spite of the many individual advances of computational neuroscience, we incompletely understand how neuronal circuits compute, and what algorithms they use to do so. I propose that the neuroscience community consider, and devote resources toward answering, these reductionist questions:

1. What do brains compute? What are the computations that process sensory information, make choices, form memories, and plan and execute actions?
2. How are computations implemented by neural circuitry?
3. Do these transforms or computations form a small canonical set?
4. Which of the many properties of nervous systems are essential for these computations, and which are merely incidental or cell-biological?

There are many *individual* findings that relate to these questions, but few general principles. What we seek are general principles of how molecular, cellular, synaptic, and network properties give rise to the information processing necessary for perception, decision making, and action. If we knew such principles, they could be described mathematically, tested, confirmed, and simulated, but except for a very few we don’t and so they can’t. There are well-known successes: canonical cortical circuitry (Douglas & Martin, 2018), cerebellum as a liquid-state machine (Yamazaki & Tanaka, 2007) and fly sensory processing (Aso et al., 2014). Two recent and elegant examples by Stevens and co-workers present fly odor encoding as a maximum-entropy locality hash and find similar coding by face recognition neurons in monkey cortex (Dasgupta et al., 2017; Stevens, 2015, 2016, 2018). Another current promising example is the assertion that the cortical microcircuit is itself a recurrent neural network (Yuste, 2018).

General, global, answers to these questions could codify important principles of neural science (Kandel et al., 2021). Such principles would include a theory of how nervous systems compute, or instead suggest that computations are ad-hoc, an an emergent property of assembling the neural (and glial) components. Although single-unit, multi-unit, local field potential, and other recordings are routinely used to probe neuronal circuitry, along with dynamical, information-theoretic, and other analyses, these techniques have in only rare cases been able to extract the computations being performed.

## The neurobiology of neural networks revisited

A lookback: the development and utilization of back-propagation in artificial neural networks (ANNs) stimulated neurobiologists to ask how biological neural nets (BNNs) computed, and to look for parallels between ANNs and BNNs. These included a much-discussed *Nature* commentary by Francis Crick (1989), a critique by Gordon Shepherd (1989), and my own *Neurobiology of Neural Networks* (Gardner, 1993), which presented then-current parallels between ANN models and BNN neuronal circuitry. In the intervening years, powerful and broadly useful, wide, recurrent, and deep convolutional ANNs using effective but non-neuromorphic architectures have been developed. Neurobiologists have advanced our understanding of the molecular, cellular, synaptic, and network complexity of BNNs. Evolutionary complexity and molecular, cellular, and network variability of BNNs reflect ethological diversity, and BNN computations may be optimized for relevant real-world features such as locality, low-dimensionality, and relevance. If we were able to discover and analyze the computations carried out by BNNs, then the ways the BNN circuits compute, adjust synaptic strength and other neural and glial properties and perform credit assignment might further enhance ANN design and performance. ANNs hewing more closely to neuronal architecture and algorithms might solve additional problems isomorphic to the ones the brain evolved to solve.

## Is neural circuitry identifiable and analyzable? Electrical circuits are.

What would enhance computational neuroscience is a schema for neural circuit analysis similar to those readily applied to circuits of electrical and electronic components. Transistors, gates, capacitors, and resistors can be purposefully assembled to form devices with defined but complex functions: half-adders, shift-registers, amplifiers, filters, oscillators, and more. A small set of principles govern such circuits’ behavior, their function can be analyzed with probes of voltage vs. time, and such recordings can be correlated with the computations or signal processing being performed. Function is readily predictable from the circuitry and the circuitry can be reverse-engineered or system-identified from its behavior (Fig. 1b; Morris & Miller, 1971.) We can’t do this; predicting the behavior of BNNs over times greater than a few seconds or applying system identification to neural circuits the same way is very difficult for any but some detectable simple neural circuit operations or calculations, including:

- Weighted summation; leaky integration; gating; winner-take-all
- Lateral/surround/directional inhibition: sharpening, gain control
- Persistence, delay, attractors, predictors for pattern completion & more
- Nonlinear A/D and D/A

The compendium of neural microcircuits by Shepherd and Grillner (2018) lays out anatomy, connectivity, function, and relevance of more than a hundred microcircuits in elegant detail, but rarely describes the computations they perform. Many, perhaps most, of these circuits have computational function or potential, but strikingly few of the descriptions show correlations between neuronal circuitry, activity, or dynamics, and what is being computed or information processing. The first (2010) edition cited ‘neurocomputing’ and ‘neuronal computation’ only once each (Ito, 2010; Wang et al., 2010) and used the word ‘neuromorphic’ in only one instance, describing fly vision as a neuromorphic circuit (Strausfeld, 2010). The second (2018) edition offers one chapter directly presenting the cortical microcircuit as a recurrent neural network (Yuste, 2018) and some explicit neuronal machine models (Ito, 2018).

By contrast, the functions of, and operations performed by ANNs seem to be emergent properties of connectivity and hidden layer properties, not readily described by any basic yet comprehensive theory.

## What components or connectivity enable neuronal computation?

If network and circuit properties and computations are evolutionarily conserved across phyla, there may be a set of canonical identifiable micro- and meso-circuits and computations, and these are likely to leverage what’s common to all or most nervous systems:

- a parts kit of differently-abled neurons and glia that vary in biochemistry, structure, connectivity, function, and integration, assembled into computational processing units,
- neurons that are post-mitotic and very long-lived, enabling persistence of informative structure,
- irregular firing and stochastic plastic synapses, straining determinism,
- alternating digital spikes with analog graded PSPs, linked by nonlinear *f*-*I* relationships
- dynamic but stable complex networks with selective balancing of inhibition, high fan-in and fan-out, and both fixed and adaptive components, and
- local processing nets linked by distant connectivity—top-down, bottom-up, and lateral—enabling both coherence and modulation

There exists a complementary set of properties that are found in some but not all nervous systems, and so are unlikely to be universally essential for computation. No single specific architecture or connectivity is essential, because multiple BNN architectures can compute and learn. What appears universal about connectomics is not a single rule for making connections, but that each circuit follows some rule. The neuromorphic question becomes what do such rules have in common. Excitatory/inhibitory balance varies greatly among different neuronal architectures. Computation is not simply an emergent property of neuron number—unless the number is very small—as flies, worms, gastropod molluscs, leeches, and others function perfectly well, including learning, with small neuron numbers. A parallel argument can be made for the number of ‘layers’ or layer-equivalent processing stages. There is of course no single canonical neuron type, as the many types of neurons each differentially express specific patterns of RNAs and proteins and form highly individual cell structures that make specific connections. Nor is there a universal neuronal microcircuit.

What is also not yet clear is which of the many common properties of neurons, synapses, and neuronal networks are necessary for computation, and which are simply cell-biological, aiding neuronal metabolism or stability. Structures or processes unlikely to mediate actual computational work performed by networks of real neurons include mitochondria, glycolysis or oxidative phosphorylation, endocytosis, Na/K ATPases, endolysosomes, and many others central to the function of non-neural as well as neural cells.

## An opposing perspective: Could it all be ad-hoc?

We seek a general theory, based firmly in neuroscience, for how nervous systems compute. The search for neuromorphic properties across diverse nervous systems posits that each of many circuit types has commonalities that give rise to the ability to process information via computation, so each of these forms of neuronal wetware can instantiate functional algorithms.

Alternatively, there may be no generality nor canonical circuitry. Neurons may have—may be—their own logic, and evolution builds ad-hoc neural networks with common components but not principles. Computability may be inherent in any neuronal circuitry, an emergent property of any circuit that transforms information using extensive connectivity and globally-informed learning rules. The diversity of neural circuit designs may just present multiple examples of evolution converging on computability, analogous to the many different ways vision has appeared and been implemented in organisms. This implies that simple summation of information, with some nonlinearities, convergence, and divergence is all that nervous systems need to do, sufficient for simple transforms such as integration. ANNs themselves suggest the possibility of no underlying algorithmic principle. In many cases, ANN function seems to be an emergent property of the connectivity matrices and hidden layer unit properties. Such multilayer nets seem to be good models in some cases: early visual and olfactory sensation, and for mapping upper to lower motor neurons, but not for many other neural computations. Thus there may well be a number of discoverable neuromorphic functional computations for which the underlying principles remain unclear.

Another counterargument says that the specific functions that different neuronal circuits perform need only to be specified by an input/output table. This is descriptive but not analytic, and like any black-box view, capable of being modeled phenomenologically only in terms of the inputs and outputs observed, disregarding the specific local circuitry or connectivity that performs the task.

There is at present insufficient evidence to accept or reject any of these explanations of neuronal function. At a symposium organized for the purpose at the 2019 Society for Neuroscience meeting (Gardner, 2019), attendees were asked if they thought neural computations and circuits were likely to be computationally conserved, and the community consensus was clear: 90% voted ‘yes’. And there is continued interest in this question. Luo (2021) called for extended examination of information-processing neuronal motifs, but he jumped from the simplest circuitry to between-region connectivity, thus bypassing the range that may yield the greatest insight. He also offered the metaphor of letters and words for neuronal assembly, but text is inherently serial and any understanding of neuronal circuitry must acknowledge the extreme parallelism. Finally, Demas et al. (2021) promoted light-beam microscopy as a tool “for discovering the neurocomputations underlying cortex-wide encoding and processing of information in the mammalian brain.”

## Summary—and a call for discussion and collaboration

Evolution conserves components below, and goals above, the levels for circuits and computations. I have presented evidence consistent with similar evolutionary conservation of a small, possibly canonical number of circuits and computations, and cited some historical interest in this idea. Electronic circuits are examples of what we would like to know. There are several strikingly common features of nervous systems that may be both conserved and computational essential. There is always a null hypothesis, and I have acknowledged the possibility that computation itself is ad-hoc in multiple areas and nervous systems, and not itself a conserved property. But we don’t know, and we should. To quote Tommy Poggio: ‘The problem of intelligence is not solved as either a scientific or engineering problem’ (personal communication). To which I suggest, let’s try the ‘scientific’—in our case, neuroscientific—and the engineering, and the math, as computational neuroscientists always do. Poggio was referring to artificial intelligence, but the question is far more interesting for biomedical, natural, intelligence.

Many of us are producing important insights toward this idea, but these are almost always focused on one neural circuit or testing one specific theory. To explore and test the concept of canonical circuits requires expanding beyond the individual-lab paradigm to a collaboration. To build on the many insights derived from single preparations, collegial efforts are needed to explore and test promising candidates for evolutionarily conserved, possibly canonical, circuits and computations. Toward this end, I call for a Neuromorphic Neural Network initiative, abbreviated **N**^3^ and pronounced ‘Encubed’. One way to start would be a focused meeting of computational and systems neuroscientists, ANN developers, and AI researchers. Each attendee would give a plenary talk advancing a potential canonical circuit or calculation (or refuting the concept). This is likely to be productive if at least half the invitees say: ‘I’m already doing that’ or ‘I’ve already solved that problem.’ This might engage the larger neuroscience community, and possibly public and private funding agencies.
